# Dr. M. J. Ely

**Published:** 1883-10

**Authors:** 


### DR. M. J. ELY.

The untimely death of Dr. Ely took place in this city October 8th. He was born April 19th, 1851, at Union Springs, Ala.; was graduated from Nashville Medical College in 1871; settled at LaFayette, Ala., and was there married to Miss Ellen Moore in 1873. Dr. Ely remained in LaFayette, prosecuting his profession, till April last, when he removed to this city, and was just becoming known as an earnest, capable man, rapidly rising in his profession, when the ruthless enemy of us all cut short his earthly aspirations.

